# T209. TESTING CORTICAL RTMS TARGETS TO IMPROVE PSYCHOMOTOR SLOWING IN SCHIZOPHRENIA AND MAJOR DEPRESSION IN A RANDOMIZED, DOUBLE-BLIND, PLACEBO-CONTROLLED TRIAL

**DOI:** 10.1093/schbul/sby016.485

**Published:** 2018-04-01

**Authors:** Sebastian Walther, Lea Schäppi, Danai Alexaki, Katharina Stegmayer, Georgios Schoretsanitis

**Affiliations:** University Hospital of Psychiatry

## Abstract

**Background:**

Psychomotor retardation is a frequent symptom of schizophrenia spectrum disorders and major depressive disorders, which hampers functional outcome. Neuroimaging studies have pointed to aberrant connectivity between cortical and subcortical components of the motor system in subjects with psychomotor retardation. Furthermore, increased neural activity was noted in premotor areas in subjects with severe motor inhibition. Interventional trials targeting aberrant brain function with noninvasive brain stimulation in this field are missing.

**Methods:**

In a randomized, sham-controlled, double-blind clinical trial we test whether three different repetitive transcranial magnetic stimulation (rTMS) protocols may ameliorate psychomotor retardation after 15 daily sessions in patients with schizophrenia spectrum disorders and patients with major depressive disorder. Randomization is performed in parallel for both diagnoses. rTMS protocols include facilitatory stimulation (15 Hz) of left dorsolateral prefrontal cortex (DLPFC), facilitatory stimulation (iTBS) of the supplementary motor area (SMA), inhibitory stimulation (1 Hz) of the SMA, and sham stimulation of the occipital cortex. Assessments are performed at baseline and every five rTMS sessions. Motor retardation is assessed with wrist actigraphy and the Salpetriere Retardation Rating Scale (SRRS). The primary outcome variable is the proportion of responders per group, with SRRS score reduction of 30% from baseline. We apply the last observation carried forward method to the intention to treat population

**Results:**

The ongoing study has enrolled 24 patients (17 SZ, 7 MDD), and 15 patients completed the study. The proportion of responders differs significantly between groups (X2 = 7.7 p = 0.05) in favor of the inhibitory SMA stimulation (83%). Repeated measures ANOVA of SRRS in all participants with LOCF indicated a significant effect of time (F = 9.6, p = 0.001), but no time x protocol interaction. However, the completer analysis indicated an effect of time (F = 14.4, p < 0.001) and a time x protocol interaction (F = 2.5, p = .05). Positive effects were also noted for fine motor performance and negative symptoms.

**Discussion:**

Inhibitory stimulation of the SMA seems to improve psychomotor retardation in these preliminary analyses. The result fits to findings of increased neural activity in premotor areas during behavioral motor inhibition in schizophrenia and major depression. Given, the effect is stable over the whole planned study population, inhibitory rTMS would become an interesting treatment for psychomotor retardation in affective and nonaffective psychoses.

